# Perivascular glial reactivity is a feature of phosphorylated tau lesions in chronic traumatic encephalopathy

**DOI:** 10.1007/s00401-025-02854-x

**Published:** 2025-02-08

**Authors:** Chelsie Osterman, Danica Hamlin, Catherine M. Suter, Andrew J. Affleck, Brian S. Gloss, Clinton P. Turner, Richard L. M. Faull, Thor D. Stein, Ann McKee, Michael E. Buckland, Maurice A. Curtis, Helen C. Murray

**Affiliations:** 1 Department of Anatomy and Medical Imaging and Centre for Brain Research, Faculty of Medical and Health Science, University of Auckland, 85 Park Road, Grafton, 1023 Auckland, New Zealand; 2 Department of Neuropathology, Royal Prince Alfred Hospital, 94 Mallet St, Camperdown, NSW 2050 Australia; 3 School of Medical Sciences, Faculty of Medicine and Health, University of Sydney, Camperdown, NSW 2006 Australia; 4 Westmead Research Hub, Westmead Institute for Medical Research, Westmead, NSW Australia; 5 Department of Anatomical Pathology, Pathology and Laboratory Medicine, Auckland City Hospital, 2 Park Road, Grafton, 1023 Auckland, New Zealand; 6 Department of Pathology and Laboratory Medicine, VA Boston Healthcare System, Boston, MA USA; 7 Department of Pathology, Boston University Chobanian & Avedisian School of Medicine, Boston, MA USA; 8 Alzheimer’s Disease and CTE Center, Boston University Chobanian & Avedisian School of Medicine, Boston, MA USA; 9 Bedford Veterans Affairs Medical Center, Bedford, MA USA

**Keywords:** Chronic traumatic encephalopathy, Neuropathology, Tau, Astrocyte, Neuroinflammation, Neurodegeneration, Brain injury

## Abstract

Chronic traumatic encephalopathy (CTE), a neurodegenerative disease associated with repetitive head injuries, is characterised by perivascular hyperphosphorylated tau (p-tau) accumulations within the depths of cortical sulci. Although the majority of CTE literature focuses on p-tau pathology, other pathological features such as glial reactivity, vascular damage, and axonal damage are relatively unexplored. In this study, we aimed to characterise these other pathological features, specifically in CTE p-tau lesion areas, to better understand the microenvironment surrounding the lesion. We utilised multiplex immunohistochemistry to investigate the distribution of 32 different markers of cytoarchitecture and pathology that are relevant to both traumatic brain injury and neurodegeneration. We qualitatively assessed the multiplex images and measured the percentage area of labelling for each marker in the lesion and non-lesion areas of CTE cases. We identified perivascular glial reactivity as a prominent feature of CTE p-tau lesions, largely driven by increases in astrocyte reactivity compared to non-lesion areas. Furthermore, we identified astrocytes labelled for both NAD(P)H quinone dehydrogenase 1 (NQO1) and L-ferritin, indicating that lesion-associated glial reactivity may be a compensatory response to iron-induced oxidative stress. Our findings demonstrate that perivascular inflammation is a consistent feature of the CTE pathognomonic lesion and may contribute to the pathogenesis of brain injury-related neurodegeneration.

**Supplementary Information:**

The online version contains supplementary material available at 10.1007/s00401-025-02854-x.

## Introduction

Chronic traumatic encephalopathy (CTE) is a progressive tauopathy. The neuropathology of CTE is defined by the perivascular accumulation of hyperphosphorylated tau (p-tau) in neurons, with or without astrocytes, deep in the cortical grey matter of the sulcal fundus (commonly referred to as the depths of the sulci) [1, 2]. This pattern of p-tau accumulation has most frequently been reported in people who have experienced repeated head impacts [3].

The distribution of p-tau pathology in CTE is a neuropathological hallmark, defining the stage of disease. In the earliest stages (“low CTE”), p-tau pathology is predominantly cortical, present as isolated, perivascular clusters at the depths of sulci. At more advanced stages (“high CTE”), p-tau pathology is widely distributed throughout neighbouring gyri and subcortical structures. The presence of astrocytic p-tau within the lesions is associated with increasing age at death [4]. The cortical perivascular p-tau lesions also evolve over time with increasing disease severity. Like Alzheimer’s disease (AD), CTE is a mixed tauopathy with neurofibrillary tangles composed of 3R and 4R tau. As CTE pathological severity increases, the predominant isoform observed within the lesion shifts from 4R towards 3R which is indicative of increased neurofibrillary tangle maturity [4–6].

Whilst p-tau is the defining feature of CTE neuropathology, non-aggregate pathologies such as neuroinflammation, axonal damage, and vascular changes are known to have pivotal roles in other neurodegenerative tauopathies such as AD and in post-traumatic sequelae, highlighting their potential for large contributors to CTE neuropathology. Furthermore, the current prevailing hypothesis of CTE pathogenesis is that head motion generates shear stress in the parenchyma concentrating at sites of structural inhomogeneity, such as the sulcal depths and around blood vessels, causing damage to the blood–brain barrier and subsequent inflammation [7, 8]. This hypothesis suggests that repeated head impacts may lead to chronic inflammation and the development of neurodegenerative p-tau pathology. However, relatively few studies have examined vascular integrity, glial reactivity or inflammation in the CTE lesion microenvironment [9].

In this study, we characterised the distribution of pathological features present within the CTE p-tau lesion areas, including neuroinflammatory and vascular changes that may better inform the current understanding of CTE pathogenesis. To achieve this, we conducted a comprehensive immunohistochemical characterisation of CTE neuropathology using tissue provided by three brain banks. We compared the pathognomonic CTE p-tau lesion area to non-lesion areas of the cortex to examine the immunohistochemical signature of CTE lesion pathology. We examined 32 markers of tissue cytoarchitecture and pathology that are relevant to both traumatic brain injury and neurodegeneration.

We incorporated antibodies against proteins previously shown to be altered in CTE cases, although not uniquely within the lesion areas, such as the microglial reactivity marker CD68 and astrocyte reactivity markers GFAP and NQO1 [10–12]. Specific combinations of antibodies that label core components of tissue architecture and pathology were also used. This included markers of neuronal and glial subtypes, reactive gliosis, blood vessels, axons and tau pathology. Due to the limited amount of tissue available and the focal nature of CTE p-tau lesions, we used a multiplex immunohistochemistry approach. Eight sequential rounds of 5-plex antibody labelling and stripping were performed, and whole-section wide-field fluorescence microscope images of each labelling round were registered to each other using cell nuclei as fiducial markers [13]. This precise alignment enabled us to create composite images of 32 antibodies on the same tissue section, establishing microscopic resolution of the whole lesion microenvironment. We conducted a qualitative and quantitative analysis of these images to identify pathological features that distinguish the CTE lesion from areas of cortical tissue without tau pathology within the same case.

## Methods

### Human brain tissue

All brain tissue was donated with the written informed consent of the next of kin and the relevant institutional approvals for brain donation, neuropathological assessment and postmortem clinical record retrieval. Three different brain banks provided the CTE cases examined in this study: eight cases from the Australian Sports Brain Bank at the Department of Neuropathology, Royal Prince Alfred Hospital, Sydney, Australia (SLHD HREC (RPAH Zone) Approval X23-0073), six cases from the UNITE Brain Bank (Boston University Medical Campus and the Veteran Affairs Bedford Institutional Review Board approval), and one case from the Neurological Foundation New Zealand Human Brain Bank (NZ HuBB) at the University of Auckland, New Zealand (Health and Disability Ethics Committee Approval 14/NTA/208) (Table 1). Cases that met the following criteria were selected by the respective brain banks: 1) presence of perivascular p-tau lesions in the superior frontal cortex; 2) contact sport as the primary head impact exposure; 3) minimal comorbid pathology. The UNITE brain bank cases were randomly selected from a list of all cases that met these three criteria. Cases were only included in the analysis if the sections that contained the pathognomonic lesion had no other aggregate pathologies present, such as alpha-synuclein Lewy bodies, TDP43 inclusions or beta-amyloid plaques. One CTE case that had minimal amyloid pathology was included, as no plaques were present in the superior frontal cortex area we examined. The primary head injury exposure for all CTE cases was contact sport, including Australian Rules Football, Rugby League, Rugby Union, Boxing, American Football, Wrestling, and Ice Hockey. Sections of the superior frontal gyrus containing at least one perivascular p-tau lesion were examined.

**Table 1:** Subject information for CTE cases included in this study

Case	Brain Bank	Age (decade)	Sex	PMD (hh:mm)	Cause of death related to cancer or cardiovascular disease^1^	Head impact exposure^2^	McKee stage	CTE severity	NIA-AA score
AU1	ASBB	50 s	M	57:00*	No	Australian Rules Football	1	Low	Not AD
AU2	ASBB	30 s	M	72:00*	No	Australian Rules Football, Boxing,	3–4	High	Not AD
AU3	ASBB	20 s	M	48:00*	No	Rugby League, Rugby Union	1–2	Low	Not AD
AU4	ASBB	40 s	M	26:00*	No	Australian Rules Football	1–2	Low	Not AD
AU5	ASBB	30 s	M	266:00*	No	Australian Rules Football	1	Low	Not AD
AU6	ASBB	50 s	M	115:00*	No	Australian Rules Football	2	Low	Not AD
AU7	ASBB	40 s	M	112:00*	No	Rugby League	3	High	Not AD
AU8	ASBB	40 s	M	157:00*	No	Rugby League	3	High	Not AD
NZ1	NZ HuBB	30 s	M	NA	No	Rugby Union	2	Low	Not AD
BU1	UNITE	50 s	M	20:45	No	American Football, Wrestling, Rugby	2	Low	Not AD
BU2	UNITE	60 s	M	17:45	Cancer	American Football, Ice Hockey, Rugby	1	Low	Not AD
BU3	UNITE	50 s	M	25:30	Cardiovascular disease	Wrestling, Rugby	3	High	Not AD
BU4	UNITE	50 s	M	18:00	NA	Boxing, Rugby	4	High	Not AD
BU5	UNITE	60 s	M	NA	No	American Football	3	High	Intermediate
BU6	UNITE	50 s	M	NA	No	Rugby	2	Low	Not AD

*Approximate PMD based on available data

^1^Cause of death has not been reported to maintain subject confidentiality unless related to cancer or cardiovascular disease as these conditions can influence the expression of inflammatory markers

^2^Primary contact sport listed first

Note: age has been reported as decade at age of death to maintain subject confidentiality

*AD* Alzheimer’s disease, *ASBB* Australian Sports Brain Bank, *NA* not available, *NZ Hubb* Neurological Foundation New Zealand Human Brain Bank, *UNITE* Understanding Neurologic Injury and Traumatic Encephalopathy Brain Bank, formerly the VA-BU-CLF Brain Bank

The diagnosis of CTE was made using the NINDS criteria [1, 2] and the severity of CTE p-tau pathology was also classified according to the second NINDS consensus criteria and the McKee staging scheme [1, 14, 15]. Briefly, tissue from the UNITE brain bank was fixed in periodate-lysine-paraformaldehyde and stored at 4 °C [14]. Tissue from the Australia Sports Brain Bank and the CTE case from the NZ HuBB was fixed in 10% neutral buffered formalin for a minimum of 2 weeks. For this study, the tissue was paraffin-embedded and sectioned in the coronal plane at a thickness of 5 μm.

For positive control labelling, an AD and a neurologically normal case were labelled and examined alongside the CTE cases. The AD and normal case were provided by the NZ HuBB, with diagnosis confirmed by an independent neuropathologist. The normal case (male, age 73) had no history of neurological abnormalities, and no other neuropathology was noted. The AD case (female, age 81) had a clinical history of dementia and was classified as having high neuropathologic change (NIA-AA score). The right hemisphere was fixed by perfusion of 10% formaldehyde in 0.1 M phosphate buffer through the cerebral arteries and was subsequently dissected into approximately 60 blocks as described previously [16]. A 0.5-cm-thick section from each block was selected for paraffin embedding. The paraffin blocks were sectioned in the coronal plane at a thickness of 5 μm using a rotary microtome (Leica Biosystems). The sections were mounted on UberC slides (Instrumec).

### Multiplex immunohistochemistry

Multiplex fluorescent immunohistochemistry was performed as previously described on 5-um-thick paraffin sections of the human superior frontal gyrus from CTE cases [13]. 5-plex labelling was achieved by combining monoclonal mouse antibodies from each available IgG subclass (IgG1, IgG2a, IgG2b, IgG3) and non-mouse hosts (rat, rabbit, chicken, and guinea pig). The eight antibody panels (Supplementary Table 1) targeted relevant antigens to map the cytoarchitecture and neuropathology related to CTE and AD. The antibody panels were designed with internal controls, whereby each antibody used in a labelling round predominantly targeted a spatially distinct sub-cellular location or tissue structure, with each antibody visualised using a different spectrally non-overlapping fluorophore. This design allowed for empirical assessment of antibody cross-reactivity and the effectiveness of antibody removal after the stripping step in between each staining round, as demonstrated in Supplementary Fig. 1. We also validated the distribution and intensity of the labelling for the antibodies labelled in rounds 2–8 matched the distribution and intensity of that seen when those same antibodies were labelled using standard single round fluorescent immunohistochemistry (Supplementary Fig. 2).

Briefly, sections were deparaffinised, rehydrated and subjected to heat-mediated antigen retrieval using 10 mM Tris/EDTA buffer pH9. The sections were then blocked with normal goat serum for 1 h and treated with TrueBlack lipofuscin autofluorescence quencher (23,007, Biotium) to reduce tissue autofluorescence. Following this, the sections were incubated overnight at 4 °C in the first-round primary antibody cocktail as per the antibody panel (Supplementary Table 1). Antibodies were diluted in 1% normal goat serum for all panels. The sections were washed with phosphate-buffered saline (PBS) and subjected to a 3-h incubation in a cocktail solution of appropriate secondary antibodies and the Hoechst nuclei counterstain (Supplementary Table 1) at room temperature. Finally, the sections were washed in PBS before being cover slipped using slowfade mounting medium.

After the first round of labelling, whole-section images were acquired with a Zeiss Z2 Axioimager with a 10X objective (0.9 NA) using MetaSystems VSlide acquisition software and MetaCyte stitching software. The Zeiss Z2 Axioimager is equipped with a Colibri 7 solid-state light source with LED lamps and the following filter sets to enable spectral separation of 6 fluorophores per round (Ex peak (nm); Em (nm)/bandpass (nm)): Hoechst 33,258 (385; 447/60), Alexa Fluor® 488 (475; 550/32), Alexa Fluor® 546 (555; 580/23), Alexa Fluor® 594 (590; 628/32), Alexa Fluor® 647 (630; 676/29), and Alexa Fluor® 800 (735; 809/81).

After imaging, the coverslips were removed from the sections by immersion in PBS at 37 °C. The antibodies were stripped from the tissue by applying 5X NewBlot™ Nitro Stripping Buffer (undiluted; LI-COR Biosciences) for 10 min at room temperature. We have previously demonstrated the efficacy of this antibody stripping protocol [13, 17, 18]. The sections were washed again in PBS, and subsequent rounds of labelling and imaging were performed as above.

For the final round of labelling, additional antigen retrieval with formic acid was performed prior to incubation with the primary antibodies (99% formic acid for 3 min at room temperature). Sequential labelling using tyramide signal amplification was used for the 4R and 3R tau antibodies as previously reported [5].

### Image registration

The images from each labelling round were extracted to individual TIFF files per channel using VSViewer (v2.1.112, MetaSystems). Image registration was performed as previously described [13]. The Hoechst labelling was present for every round of labelling and imaging, and nuclei were used as the intrinsic markers for image registration between rounds. We used a custom-designed registration code in Python to automatically register the nuclei images. The Hoechst images were pre-processed by applying a 50-pixel rolling background subtraction and a 5-pixel median filter to smooth out any nuclei staining fluctuations. The processed Hoechst images were registered with each other using an AKAZE affine registration and a transformation matrix was extracted for each image set. The transformation matrix was applied to all the individual images for the round.

### Quantitative image analysis

All measurements were made using ImageJ (v1.54f; National Institutes of Health, Bethesda, MD) on 8-bit grayscale TIFF images.

An overlay of the smooth muscle actin (SMA), NeuN, and AT8 images was used to identify lesion vessels in CTE sections. Lesion vessels were defined according to the NINDS criteria for the CTE pathognomonic lesion [1, 2]. Briefly, the lesion area was defined by the presence of AT8 labelling around an SMA + blood vessel at the depth of the cortical sulcus. In cases where AT8 p-tau pathology was diffuse, the SMA + vessel(s) with the most concentrated accumulation of p-tau were selected. If multiple distinct lesion vessels in the same sulci were present, all were included where possible and if more than one distinct lesion was present in different sulci of the same case, all lesions were analysed. Up to three non-lesion SMA + vessels in the same cortical layer were selected in the neighbouring gyral wall at least 2 mm from the lesion vessels. A 1 mm^2^ region of interest was defined on the image stack containing all 32 marker channels, with the SMA + vessel positioned in the centre of each ROI. To improve image segmentation, the autofluorescence channel image was subtracted from each marker image in the stack. The mean grey value of the background staining was measured in a 50 pixel^2^ area for each image. The threshold tool was used to create binary masks of positive labelling for each marker. The lower threshold limit was set at 10 grey values above the background mean grey value, and the upper limit was set at 255, the maximum for 8-bit images (Supplementary Fig. 3). The binary masks were inspected by two observers (H.M. and C.O.) to ensure only positive labelling was consistently segmented across cases. The area of the binary masks was measured and normalised to the region of interest area to give the percentage area of the image labelled. For each case, the mean percentage area of each marker in the lesion and non-lesion regions was calculated.

Sholl analysis was conducted on the lesion and non-lesion region of interest images. For each region of interest image, a 50-µm diameter circle selection was placed over the vessel lumen. Concentric circle selections spaced 20 µm apart were then created around the centre circle selection up to 300 µm from the vessel lumen. The area of each marker’s binary mask was measured within each concentric ring and the area of labelling was normalised to the area of the ring to give the percentage area of the ring labelled. The values were normalised to the maximum value of the dataset for that case. The mean ± standard error of the mean (SEM) normalised area of labelling for each marker was plotted against the distance from the vessel.

### Statistical analysis and data visualisation

Our dataset comprised values of the mean percentage of labelling for all 32 markers in lesion and non-lesion areas for each case. Statistical analysis and graphing were performed using GraphPad Prism (v10.1.2). We first assessed the data for normality and equality of variance to determine the appropriate statistical tests. A parametric paired *t* test was used to test for differences in marker labelling area between lesion and non-lesion areas where the data fit the assumptions of normal distribution and equality of variance. If the data did not fit these assumptions, then a non-parametric Wilcoxon test was performed. Subsequently, the -log10 of the *p* value and the log2 fold change of the difference between areas were calculated for each marker and visualised using a volcano plot.

### Gene expression correlates

The relative abundance of mRNA transcripts of reactive astroglial markers in CTE lesions was obtained from spatial transcriptomics analyses performed as part of another study by author C.S. This study performed Visium (10 × Genomics) as per the manufacturer’s instructions for FFPE tissue to profile discrete CTE lesions from three of the cases used in our study (AU2, AU6, AU7); The CTE lesions profiled were obtained from similar brain regions but were not the same lesions examined using multiplex immunohistochemistry. Spatial gene expression heatmaps were generated for each of the differentially expressed astrocyte reactivity markers (NQO1, CHI3L1, GFAP, AQP4, FTL) in Loupe browser with reference to AT8 p-tau immunohistochemistry.

## Results

### Tangles within the CTE lesion have a mature immunophenotype

**Fig. 1:**
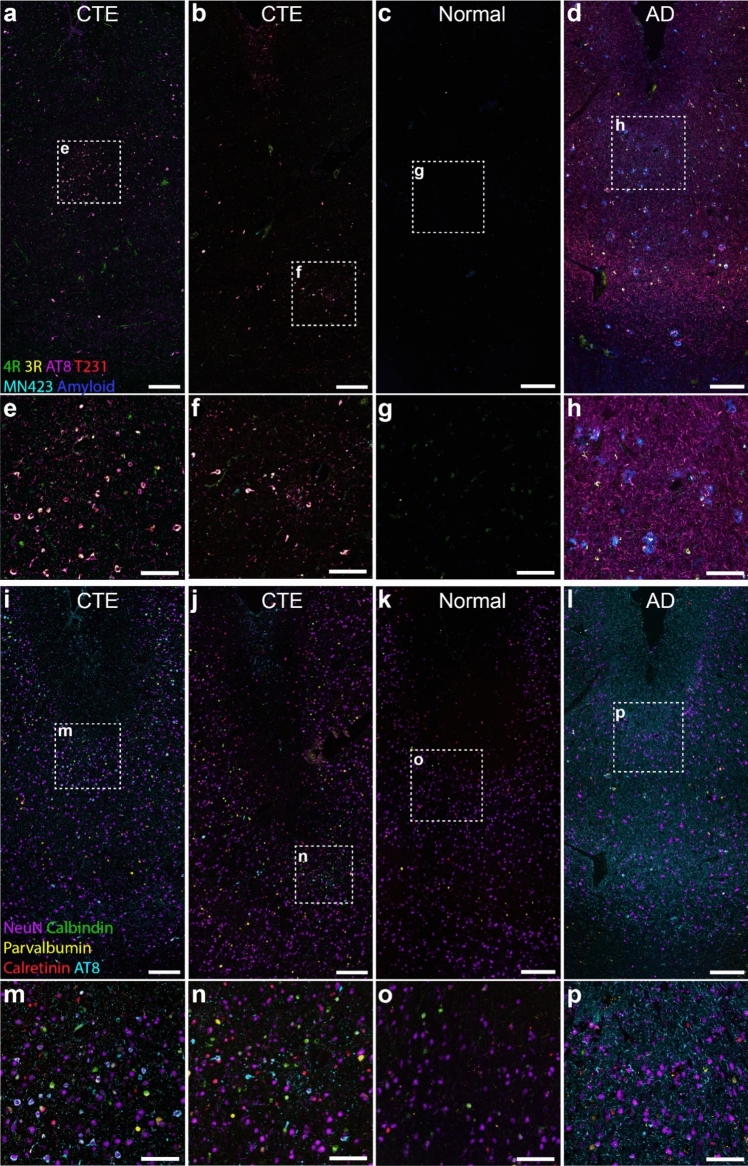
Overview of multiplex labelling for tau and neuronal markers in the frontal cortex of CTE cases. Overview of the area at the depth of the cortical sulcus containing a p-tau lesion in two CTE cases (AU2 and AU7, both high-stage CTE) from the Australia Sports Brain Bank (**a–b, e–f**), and the corresponding area in a representative neurologically normal **(c, g**) and AD case (**d, h**). Multiplex labelling for tau pathology markers 4R, 3R, p-tau202-205 (AT8), p-tau231 (T231), MN423 and beta-amyloid are presented (**a-d**), alongside a higher magnification of the lesion vessel and comparable regions in the AD and normal cases indicated by the dotted box (**e–h**). Neurofibrillary tangles labelled for 4R, 3R, AT8, and T231 within the perivascular p-tau lesion in CTE cases (**a-b, e–f**). The AD case also showed extensive neurofibrillary tangles and fibres that were labelled for 4R, 3R, AT8, and T231 (**d, h**). Tau labelling was absent in the neurologically normal case (**c, g**). Multiplex labelling for neuronal subtype markers NeuN, calbindin, calretinin, and parvalbumin are presented with AT8 (**i-l**), along with higher magnification of the regions indicated by the dotted box (**m-p**). The perivascular tau-positive cells were predominantly located in layer II of the cortex and co-labelled with the neuronal marker NeuN, but not with the interneuron markers calbindin, calretinin, or parvalbumin. Scale bars: 250 µm **(a-d, i–l)**; 100 µm,** (e–h, m–p)**

The CTE tissue sections examined in this study contained focal accumulations of p-tau within neurons and/or astrocytes around blood vessels at the depths of the cortical sulci. Using multiplex IHC, we qualitatively assessed the co-labelling of multiple proteins on the same tissue section (Fig. 1a–p). We first investigated the composition of tau neurofibrillary tangles within the CTE lesions using a panel of tau antibodies that we previously showed to be associated with tangle maturity in AD [5]. The neurofibrillary tangles within the CTE lesion co-labelled with antibodies against p-tau202-205 (AT8), p-tau231 (AT180), 4R isoform, and 3R isoform, but not for C-terminus cleaved tau (MN423, Fig. 1a–b, e–f). Whilst no neurofibrillary tangles were observed in the normal case (Fig. 1c, g), the same labelling pattern seen in CTE was observed for neurofibrillary tangles within the AD case (Fig. 1d, h). The AD case showed extensive p-tau202-205 and p-tau231 labelled neurites. Neurofibrillary tangles and neurites in the CTE cases and AD case were labelled with ubiquitin (Fig. 1i, j, l, m, n, p). Overlay of p-tau202-205 and neuronal markers showed that whilst the CTE lesions were deep in the grey matter, distant from the pial surface, they were most frequently located within layer II of the cortex. We did not observe neurofibrillary tangles within cells that were labelled for the interneuron markers calbindin, calretinin or parvalbumin in the CTE cases (Fig. 1m–n).

### CTE lesions show focal perivascular astrocyte reactivity

**Fig. 2:**
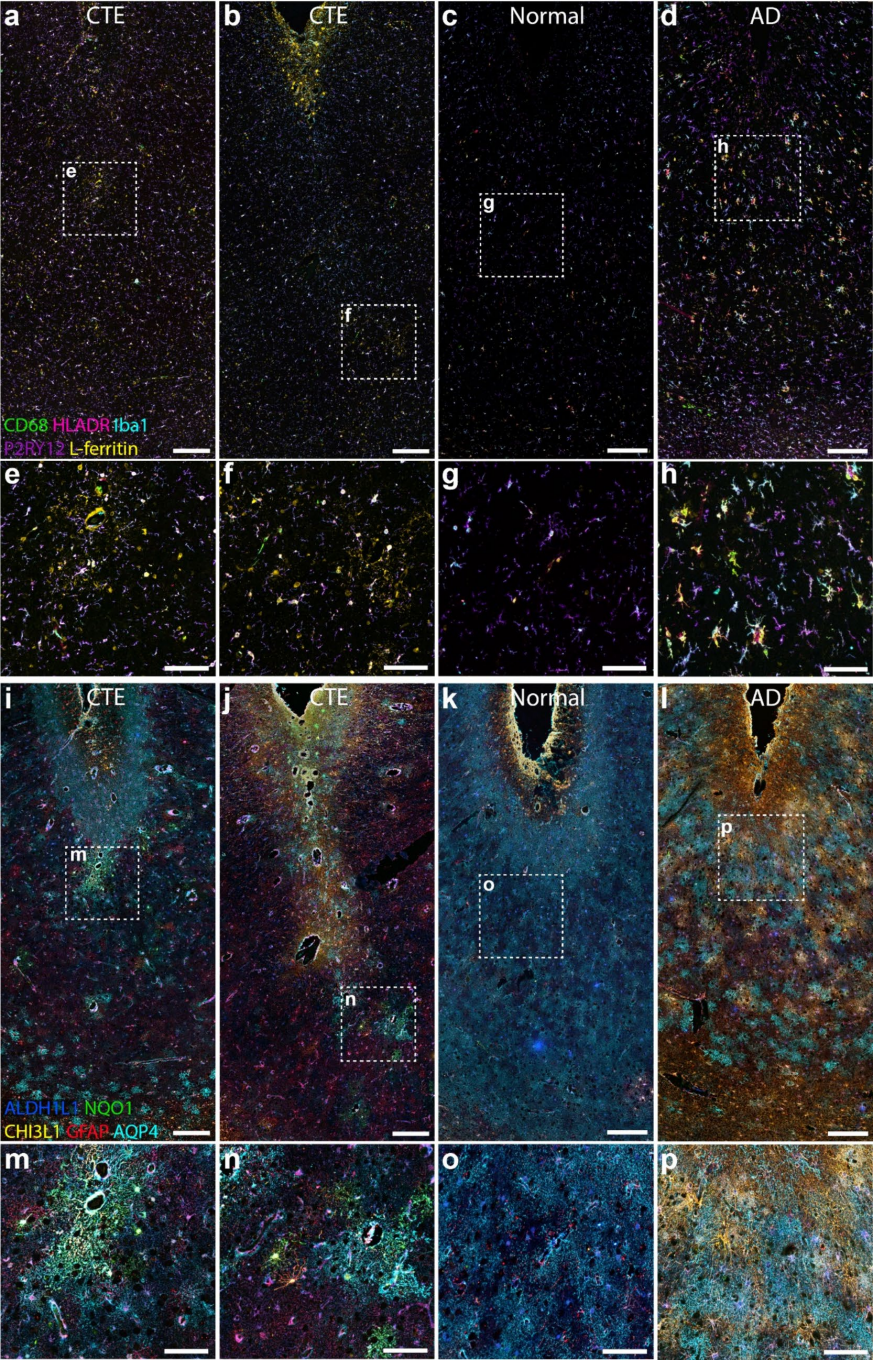
Overview of multiplex labelling for markers of reactive glia in the frontal cortex of CTE cases. Multiplex labelling of reactive microglia (**a–h**) and astrocytes (**i-p**) within the depth of the cortical sulcus containing a p-tau lesion in two CTE cases from the Australia Sports Brain Bank (**a–b, e–f**) and the corresponding area in a representative normal (**c, g**) and AD case (**d, h**). Reactive microglial markers CD68, HLA-DR and L-ferritin, and homeostatic microglial markers Iba1 and P2RY12 are presented (**a–d**) alongside higher magnification of the lesion vessel and comparable regions in AD and normal cases indicated by the dotted box (**e–h**). CTE cases show elevated immunoreactivity for L-ferritin but comparable labelling for all other microglial markers compared to normal cases. Reactive astrocyte markers NQO1, CHI3L1, and GFAP, homeostatic astrocyte marker ALDH1L1, and astrocyte water channel marker AQP4 are presented (**i-l**) alongside higher magnification of the lesion vessel and comparable regions indicated by the dotted box (**m–p**). Immunoreactivity for reactive astrocyte markers was increased around the lesion vessels in CTE cases, compared to normal cases and the more ubiquitous distribution seen in AD cases. Scale bars: 250 µm **(a–d, i–l)**; 100 µm,** (e–h, m–p)**

Our previous systematic review identified neuroinflammation as a prominent feature in CTE [9]. Therefore, we examined microglial and astrocyte reactivity markers in the CTE lesion sulci (Fig. 2a–l). The antibody panels targeted proteins that have a role in inflammatory processes within the human cortex. We examined HLA-DR, CD68 and L-ferritin as markers of microglial reactivity. Qualitatively, HLA-DR and CD68 labelling area and intensity (immunoreactivity) in the CTE cases were low, appearing comparable to that seen in the neurologically normal case (Fig. 2a–h). However, L-ferritin immunoreactivity was widespread and observed in all glial cells across the grey matter in CTE cases (Fig. 2a–b, e–f). The L-ferritin immunoreactivity had a predominantly astrocytic morphology and was most intense around the p-tau lesion vessels and in the subpial layer at the sulcal depth. The immunoreactivity of homeostatic microglial markers Iba1 and P2RY12 appeared to be evenly distributed across the grey matter in the CTE cases (Fig. 2e–h).

We examined CHI3L1, GFAP and NQO1 as markers of astrocyte reactivity (Fig. 2i–l). The immunoreactivity of these markers was increased focally around blood vessels in CTE cases, with the highest intensity labelling observed around the lesion (Fig. 2i–j, m–n). This was in contrast with the more ubiquitous distribution seen in the AD case and relative absence in the normal case. The immunoreactivity of the homeostatic astrocyte marker ALDH1L1 appeared to be ubiquitously distributed across the grey matter in the CTE cases. The distribution of aquaporin IV (AQP4) immunoreactivity was patchy and heterogeneous across and the CTE cases; however, AQP4 immunoreactivity was consistently increased around all lesion vessels compared to the surrounding grey matter.

Markers of axons, including CNPase, neurofilament light, and myelin basic protein, were ubiquitously expressed across the CTE cases, with no obvious difference in immunoreactivity noted around p-tau lesion vessels compared to adjacent areas (Supplementary Fig. 4a–b). Similarly, no differences in immunoreactivity were seen for blood vessel markers (collagen IV, UEA lectin, smooth muscle actin) or the neutrophil marker myeloperoxidase between CTE lesion and non-lesion areas (Supplementary Fig. 4e–h).

**Fig. 3:**
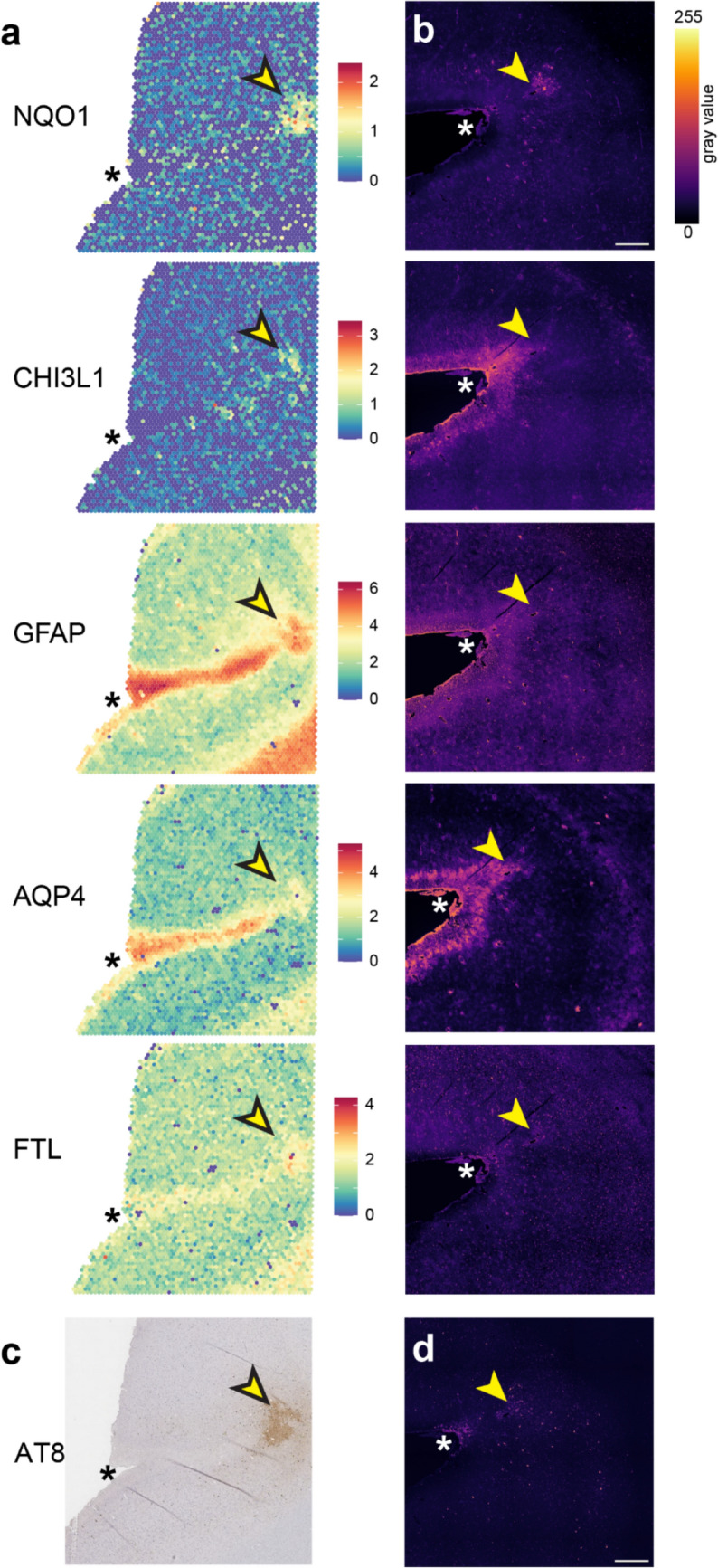
RNA and protein distribution for reactive gliosis markers in CTE lesion sulcus. **a** Spatial gene expression heatmaps generated from the Visium spatial transcriptomics data illustrates the mRNA distribution for NQO1, CHI3L1, GFAP, AQP4 and FTL (L-ferritin) within a lesion sulcus for CTE case AU6 from the Australia Sports Brain Bank. **b** Immunohistochemistry was performed for the corresponding protein in (a) on a different lesion sulcus from the same case. The p-tau (AT8) labelling for the same tissue sections is shown at the bottom **(c–d)**. Scale bar: 200 µm. In all three cases, focal increases in NQO1, CHI3L1, GFAP, AQP4 and FTL (L-ferritin) mRNA and protein expression are seen in cortical layer 1, the white matter and within the lesion area (yellow arrow) where AT8 tau is highly concentrated. The sulcal border is marked with an asterisk

Three of the CTE cases from the Australia Sports Brain Bank (AU2, AU6, and AU7) had undergone spatially resolved transcriptomics analysis as part of a different study. We used this data to interrogate the mRNA expression of astroglial markers within lesion sulci (Fig. 3a, Supplementary Fig. 5). This data was collected from a different p-tau lesion sulcus to the one examined using multiplex immunohistochemistry (Fig. 3b). The mRNA (Fig. 3a) and protein expression (Fig. 3b) for NQO1, CHI3L1, GFAP, AQP4 and ferritin light chain (FTL) were increased specifically within the regions where the AT8 tau immunoreactivity was most concentrated (Fig. 3c–d). In addition, increased mRNA and protein expression of GFAP, CHI3L1 and AQP4 was observed in cortical layer 1 relative to surrounding areas in all three CTE cases (Supplementary Fig. 5).

**Fig. 4:**
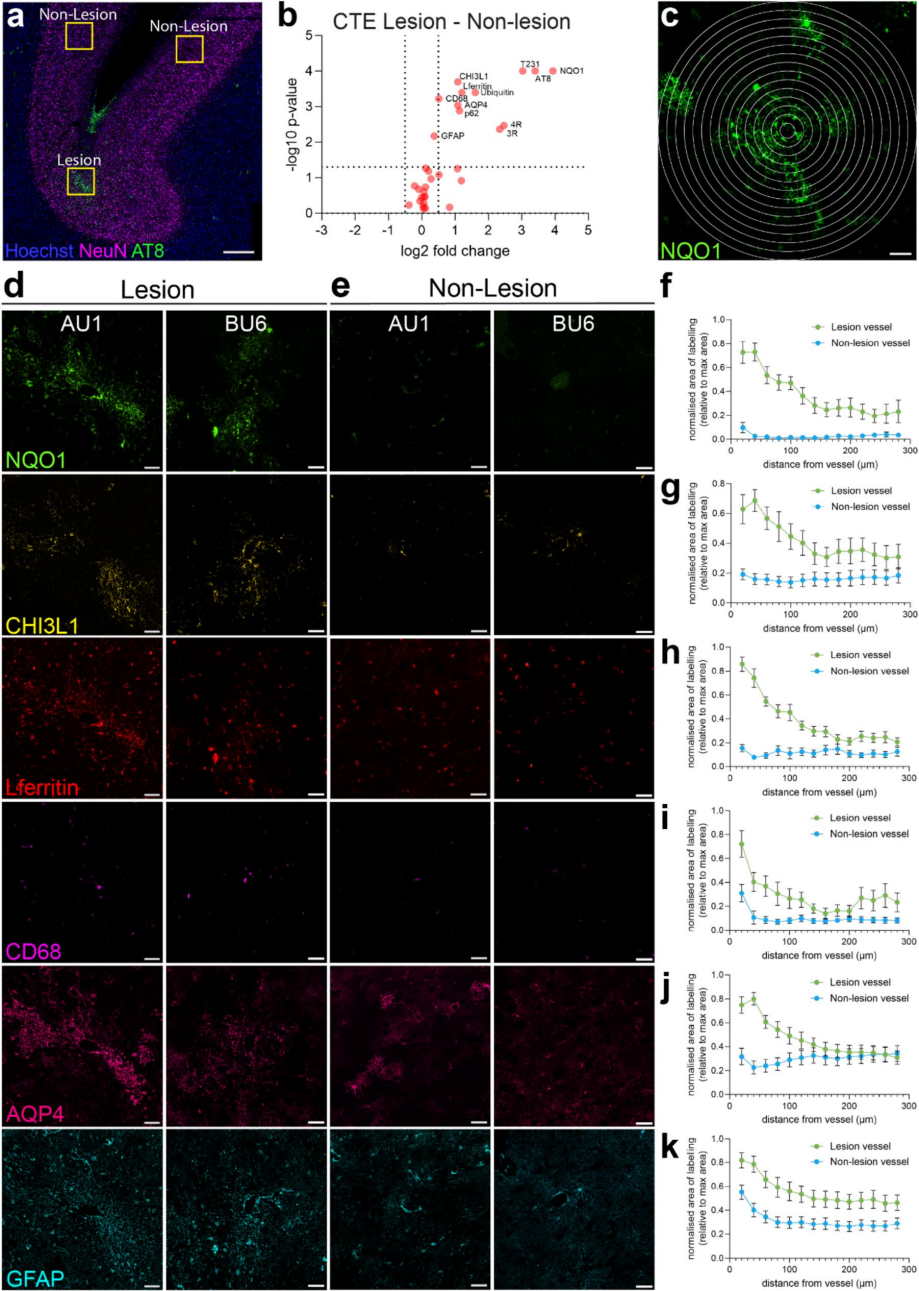
Differentially expressed markers in CTE lesion areas relative to non-lesion areas. **a** The area of labelling for each marker was measured within a 1mm^2^ region around the p-tau-positive lesion vessel in the sulci and p-tau-negative non-lesion vessels in the adjacent gyrus. Scale bar 1 mm. **b** Volcano plot illustrating differentially expressed markers between the lesion and non-lesion areas. Paired *t* tests (parametric) or Wilcoxon tests (non-parametric) were conducted to assess the statistical significance of the difference in the area of positive labelling between lesion and non-lesion regions of interest. Vertical dashed lines indicate the log2 (Fold change) cut-off of 0.5 (twofold change). Horizontal dashed lines indicate − log10 (p value) cut-off at 0.05. Each red dot represents one marker, with the mean log2 fold change between lesion and non-lesion regions across all CTE cases plotted. The labelled red dots are lesion-associated differentially expressed markers. **c** An example of the Sholl analysis. Concentric rings radiated from the vessel lumen with 20 µm spacing. Scale bar 50 µm **(d–e)** Representative images of the labelling for differentially expressed glial reactivity markers in lesion **(d)** and non-lesion **(e)** regions from two CTE cases (AU1, BU6) provided by different brain banks. Scale bars: 50 µm. **f–k** Graphs of the Sholl analysis for lesion and non-lesion vessels, where the area of labelling for each marker was measured within concentric rings up to 300 µm from the vessel lumen. The area of labelling was normalised to the area of the ring, and the values were normalised to the maximum value of the dataset for that case. The mean ± SEM normalised area of labelling for each marker was plotted against the distance from the vessel

To support our qualitative observations of focal glial reactivity around CTE p-tau lesion vessels, we quantitatively screened for differentially expressed proteins between the lesion and non-lesion areas within CTE cases. To do so, we measured the area of immunolabelling within a 1mm^2^ region around the p-tau-positive lesion vessel in the sulci and p-tau-negative non-lesion vessels in the adjacent gyral walls for each marker (Fig. 4a).

Consistent with our qualitative observations, increased glial reactivity was observed in CTE lesion areas with a significantly higher percentage area labelled for GFAP (p = 0.0067), CHI3L1 (p = 0.0002), NQO1 (p < 0.0001), AQP4 (p = 0.0009), L-ferritin (p = 0.0004) and CD68 (p = 0.0006) compared to non-lesion vessels (Fig. 4b, Supplementary Table 2). Of these markers, NQO1 showed the greatest difference in the area coverage between lesion and non-lesion vessels. There was no significant difference in the area labelled for the homeostatic markers ALDH1L1 (p = 0.514), Iba1 (p = 0.1813) and P2RY12 (p = 0.208) between the lesion and non-lesion areas (Supplementary Table 2). Consistent with our definition of CTE lesion vessels being p-tau-positive, the area labelled for markers of tau (3R, 4R, AT8 and T231) and ubiquitination (ubiquitin, p62) was significantly increased in the lesion areas compared to non-lesion areas (Fig. 4b, Supplementary Table 2).

We further analysed the perivascular localisation of the differentially expressed glial markers using a Sholl analysis (Fig. 4c). Qualitatively, we observed that the immunoreactivity for NQO1, CHI3L1, L-ferritin, CD68, AQP4 and GFAP was focally concentrated around the SMA + vessels in the lesion areas but diffusely distributed in the non-lesion areas (Fig. 4d–e). The Sholl analysis confirmed that for the lesion areas, the percentage area of labelling for these markers was highest within the first 40 µm from the vessel lumen and declined as the distance from the vessel increased (Fig. 4f–k). In the non-lesion areas, the percentage area of labelling for NQO1, CHI3L1, L-ferritin and AQP4 did not change as the distance from the vessel increased, indicating the labelling was not perivascular (Fig. 4f–k). GFAP and CD68 showed an increased percentage area of labelling within the first 20–40 µm from the vessel lumen in non-lesion areas, indicating the distribution of these markers was perivascular in both lesion and non-lesion areas (Fig. 4f–k).

We also investigated whether the percentage area of labelling correlated with age at death or postmortem delay for each of the markers we investigated. There was a significant positive correlation between age at death and the percentage area of labelling for HLA-DR and ALDH1L1 in both lesion and non-lesion areas (Supplementary Fig. 6). There were no significant correlations between postmortem delay and area of labelling for any marker in the lesion and non-lesion areas (data not shown). We identified significant differences between low and high-stage CTE cases for the percentage area labelled for Iba1 and ALDH1L1 in the lesion and non-lesion areas and for ubiquitin in lesion areas only (Supplementary Fig. 6).

### Astrocytes express NQO1 and L-ferritin within the CTE lesion areas

**Fig. 5:**
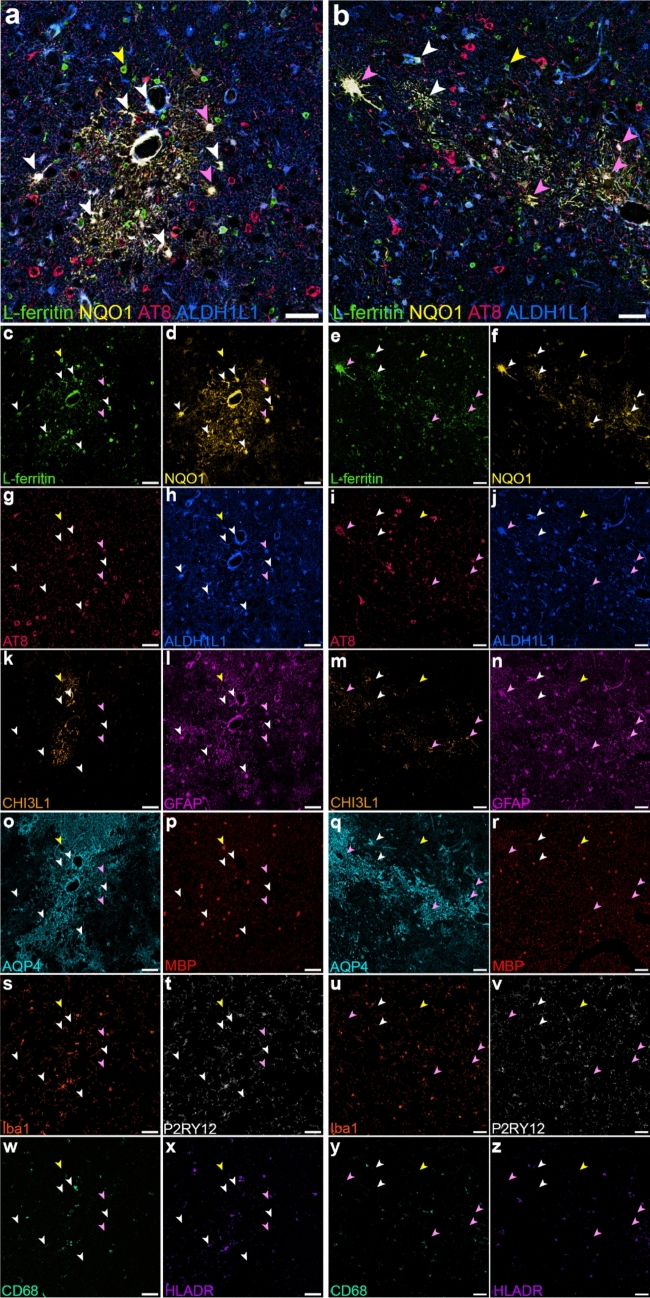
NQO1 and L-ferritin co-labelling in astrocytes within the CTE lesion. Images of the p-tau lesion from CTE case AU2 (**a**) and AU1 (**b**) illustrating co-labelling for glial reactivity markers L-ferritin (**c, e**) and NQO1 (**d, f)**, with AT8 tau (**g, i**, pink arrows) and the homeostatic astrocyte marker ALDH1L1 (**h, j**, white and pink arrows) and L-ferritin+ NQO1+ cells also co-labelled with GFAP (**l, n**), but not CHI3L1 (**k, m**) or AQP4 (**o, q**). A subset of L-ferritin cells also co-labelled with myelin basic protein (**p, r,** yellow arrows) and a different subset co-labelled for the microglial markers Iba1 (**s, u**), P2RY12 (**t, v**), CD68 (**w, y**), and HLA-DR (**x, z**). Scale bars 50 µm

Finally, as NQO1 and L-ferritin were the glial reactivity markers with the greatest difference in percentage area labelled between CTE lesion and non-lesion vessels, we further examined the phenotype of cells expressing these proteins within the lesion area. We found that L-ferritin and NQO1 frequently co-labelled on cell soma and processes (Fig. 5, white and pink arrows). L-ferritin+ NQO1+ cells co-labelled with the homeostatic astrocytic marker ALDH1L1 and the reactive astrocyte marker GFAP, although not all were labelled for CHI3L1 or AQP4. A subset of L-ferritin+ NQO1+ cells contained AT8 (p-tau202-205, Fig. 5, pink arrows). L-ferritin, but not NQO1, was also observed to co-label with the microglial markers Iba1 and P2RY12, and the oligodendrocyte marker myelin basic protein (Fig. 5, yellow arrows), indicating this protein is expressed by multiple glial populations within the CTE lesion.

## Discussion

Our findings highlight the role of perivascular glial reactivity in the pathogenesis of CTE. Non-aggregate pathologies such as neuroinflammation, axon injury and vascular damage are observed in other neurodegenerative tauopathies such as AD and as part of the secondary neurodegeneration after traumatic brain injury [7, 19–21]. Therefore, we sought to investigate whether markers of these processes were altered in the CTE lesion microenvironment compared to non-lesion areas. We characterised a broad range of pathological changes using multiplex immunohistochemistry to label 32 antibodies on the same tissue section. We identified that focal perivascular neuroinflammation was a feature of the p-tau lesion areas in CTE cases.

### Focal glial reactivity in CTE lesions

Glial reactivity mediates the neuroinflammatory response to disease and injury and is a hallmark feature of several neurodegenerative and neurological diseases [22–26]. Whilst glial reactivity is complex, the most well-characterised immunohistochemical markers include GFAP in astrocytes, and CD68 and HLA-DR in microglia and/or monocytes [27–31]. Other markers include the astrocyte-secreted glycoprotein CHI3L1, the multifunction stress protein NQO1, and the iron storage protein L-ferritin [32–35].

We identified CHI3L1, GFAP and NQO1 immunoreactivity around cortical blood vessels in CTE, which was especially concentrated around p-tau lesion vessels compared to non-lesion areas. Of note, the area labelled for the constitutively expressed astrocyte marker ALDH1L1 was not significantly different between CTE lesion and non-lesion areas suggesting no change in astrocyte density. Given the neuroinflammatory role of GFAP [36–39], CHI3L1 [40–42] and NQO1 [43–46], their increased coverage around the lesion vessels is an indication of a vessel-associated, astrocyte inflammatory profile in CTE. To date, there have been very few studies characterising astrocyte reactivity in CTE. Of those studies, CHI3L1 gene expression was increased in subpopulations of astrocytes that also express other inflammatory genes [47] and overall grey matter NQO1 expression was found to increase with CTE severity and p-tau pathology [11]. Increased GFAP immunoreactivity at the grey–white matter interface in CTE cases relative to controls has been reported [48, 49]; however, in the grey matter of CTE cases, previous studies show an increased intensity of GFAP labelling, but with no associated change in the area of labelling or the number of GFAP + cells compared to controls [10, 48, 49]. Our study differs in that we specifically examined sulci with confirmed p-tau lesions and no comorbid beta-amyloid, alpha-synuclein, or TDP-43 pathology. Our multiplexed labelling using a range of markers indicates that astrocyte reactivity is a key feature of the CTE pathognomonic lesion. Future studies with greater case numbers are required to characterise the profile of reactive astrocytes within the CTE lesion, and how age and stage of disease influence their phenotype. This is particularly important as CTE lesions evolve over time. Previous studies have demonstrated that astrocytic p-tau is associated with increasing age at death [4, 50, 51] and glial reactivity is influenced by increasing age [52–54]. Whilst our study did not examine how astrocyte reactivity markers relate to astrocytic p-tau accumulation in the CTE lesion, future studies examining this relationship will contribute to our understanding of how CTE lesions mature.

HLA-DR and CD68 are microglia and monocyte proteins classically associated with microglial reactivity due to their role in antigen presentation and phagocytosis, respectively [29, 30, 55, 56]. L-ferritin is an iron storage protein upregulated in dystrophic microglia under normal ageing and disease conditions [34, 35, 57, 58]. Quantitatively, CD68 and L-ferritin were increased around p-tau lesion vessels relative to non-lesion vessels in CTE. However, L-ferritin expression was not exclusive to microglia in CTE cases, with expression identified in astrocytes and oligodendrocytes based on co-labelling with ALDH1L1 and myelin basic protein respectively. Furthermore, HLADR and the homeostatic microglia markers IBA1 and P2RY12 were not significantly altered within lesion areas which suggests glial reactivity in CTE lesions is predominantly driven by astrocytes rather than microglia. This contrasts with the findings of Cherry et al. [10, 12], where macrophage recruitment was observed around lesion vessels, with increased numbers of Iba1+ TMEM119- cells compared to non-lesion vessels. Similarly, they reported an increased number of CD68+ microglia in CTE cases with increasing disease severity compared to control cases. The differences in our findings are likely related to the different measures we assessed (area of labelling vs cell number) and the lack of comorbid pathology in the CTE cases we examined. Our future priorities are to use this multiplex dataset to further characterise microglial immunophenotype populations in CTE.

### Oxidative stress in CTE lesions

Our multiplexed labelling revealed the co-expression of NQO1, L-ferritin and p-tau within astrocytes in the CTE lesion area. p-tau pathology is primarily neuronal in CTE; however, astrocytic p-tau pathology is a common feature of CTE lesions in older cases [1, 2, 4, 50, 51], and a growing body of evidence is increasingly implicating astrocytic involvement in post-traumatic injury disease processes. This includes a range of structural and functional changes in astrocytes that promote chronic neuroinflammation and blood–brain barrier disruption in all neurodegenerative diseases [59–62]. Furthermore, studies have identified that astrocytes preferentially engulf neuronal synapses containing aberrant tau oligomers in AD, which may contribute to p-tau accumulation within astrocytes [63, 64]. Our multiplexed labelling shows that astrocytic p-tau pathology in CTE is often found within astrocytes expressing the markers NQO1 and L-ferritin, proteins associated with protective responses to oxidative stress [65–67]. This suggests that the astrocytic response to oxidative stress may be involved in the process of p-tau accumulation.

Oxidative stress arises in response to an imbalance between the production and detoxification of free radicals, resulting in the formation of toxic species that impair cellular functions [68]. Oxidative stress has been implicated in the progression of several neurodegenerative diseases [69–71] and as a key factor in the sequelae of traumatic brain injury [72–74]. However, the role of oxidative stress in CTE is relatively unknown, with only one study that identified a CTE-specific enrichment of NQO1 within astrocytes associated with increasing p-tau pathology and CTE severity [11].

NQO1 is a multifunction stress protein expressed in astrocytes with key roles in the detoxification of reactive oxygen species. Under physiological conditions, basal expression of NQO1 is low; however, under neuroinflammatory conditions, NQO1 expression increases as a protective response to increasing free radicals [46, 65]. We identified focal expression of NQO1 within astrocytes surrounding the p-tau lesion vessels in CTE, indicating that oxidative stress may be a key feature of the CTE lesion microenvironment. Our observation that NQO1+ astrocytes also express L-ferritin suggests that oxidative stress may be driven by the presence of iron in the parenchyma due to blood–brain barrier damage.

L-ferritin is an important iron storage protein that acts to sequester free iron in nontoxic forms, mitigating iron-induced oxidative stress [34, 66]. Under physiological conditions, L-ferritin expression is largely restricted to microglia and oligodendrocytes with minimal involvement of astrocytes [75]. However, ferritin positive astrocytes have been identified with increasing age [76] and in amyotrophic lateral sclerosis [57]. Furthermore, hypoxia and reoxygenation after ischaemia increase L-ferritin synthesis in cultured astrocytes [77], whilst direct exposure to heme-bound iron increases ferritin synthesis and protects cultured astrocytes from iron-induced oxidative stress [78]. Therefore, the identification of L-ferritin+ astrocytes around CTE lesions, in combination with increasing NQO1 expression, suggests astrocytes may be mounting a compensatory response to mitigate iron-induced oxidative stress arising from a compromised blood–brain barrier. Whether these changes promote astrocyte p-tau accumulation or are instead a consequence of astrocyte p-tau accumulation remains to be determined.

### Blood–brain barrier damage

Given the perivascular distribution of astrocyte reactivity, the role of astrocytes in blood–brain barrier maintenance [79], and previous literature demonstrating blood–brain barrier abnormalities in CTE [80–83] and repeated head injuries [84, 85], we hypothesised glial reactivity in CTE may be a response to microvascular injury from repeated head trauma and subsequent leakage of vascular components into the parenchyma. Whilst we included markers of vascular structure in our antibody panel, including collagen IV for basement membrane, UEA1 lectin for endothelial cells, and smooth muscle actin for arterioles, we did not identify changes in the area coverage of these proteins. However, we did not examine markers of tight junction integrity or vascular leakage which may be implicated in CTE [81, 86]. Furthermore, subtle changes in the expression of these proteins may be better characterised with measurements of fluorescent intensity rather than the area of labelling as measured in this study. Overall, whilst we did not identify any overt changes in vasculature markers in CTE cases, further characterisation of vascular integrity and blood–brain barrier leakage in CTE is required.

The water channel AQP4 expressed on astrocyte end feet around the blood–brain barrier was differentially expressed between CTE lesion vessels and non-lesion vessels. AQP4 is essential for water transport in and out of blood vessels [87, 88]. Altered AQP4 expression and polarisation are increasingly reported in neurodegenerative diseases, with decreasing AQP4 vessel polarisation hypothesised to contribute to impaired waste clearance and pathogenic protein accumulation [89–92]. We found that lesion vessels in CTE demonstrated a focal increase in AQP4 immunoreactivity compared to the adjacent parenchyma. This increased AQP4 expression has been previously reported in CTE in a doctoral thesis [93] with p-tau lesion vessels showing increased AQP4 compared to p-tau negative non-lesion vessels, and in individuals exposed to blast injury [94]. Given the role of AQP4 in water homeostasis, the increased localization of AQP4 to the lesion vessel may reflect a compensatory response to vascular damage in CTE to resolve trauma-induced oedema. However, it is important to note that the distribution of AQP4 throughout the grey matter was heterogenous in all cases. This is likely due to age-related [92] and/or psychiatric confounding effects [95], both of which have been associated with changes in AQP4 polarisation.

### Neuronal pathology in CTE

Our multiplex immunohistochemistry panels included markers of neuronal subtypes, axonal proteins and tau. Neurofibrillary tangles in CTE have been extensively examined in previous studies and shown to be labelled with 3R, 4R and p-tau202-205 (AT8) [4, 96, 97]. We also found the neurofibrillary tangles within CTE lesions co-labelled for these markers, as well as p-tau231 (AT180), but not for C-terminus cleaved tau (MN423). In line with our previous examination of neurofibrillary tangles in AD [5], these findings suggest that neurofibrillary tangles within the CTE lesion have a relatively mature immunophenotype and morphology. However, further analysis of tau antibody co-labelling is required to assess how tangle maturity varies between the lesion and non-lesion areas of the cortex. Our qualitative analysis of tau co-labelling with neuronal markers indicated that CTE lesions were predominantly located in layer II of the cortex. We also noted that tau+ NeuN+ cells did not express common interneuron markers such as calbindin, calretinin and parvalbumin, indicating interneuron populations are relatively unaffected in lesion areas. In contrast with previous studies that describe reduced myelination in the white matter of CTE cases, we did not observe any overt loss of immunoreactivity for myelin basic protein, CNPase, or neurofilament light in CTE lesion areas [98, 99]. It is possible that more sensitive measures, such as fluorescent intensity, may be required to identify more subtle changes in axon integrity in the grey matter.

## Limitations

There are several limitations to our study. We used CTE tissue from three different brain banks to ensure our findings were consistent across a range of CTE cases. However, as each brain bank has different processes for receiving and processing tissue, there is significant variability in postmortem delay and tissue fixation methods between the CTE cases examined in this study.

As the objective of our study was to identify pathological features that were specific to the CTE p-tau lesions, we excluded cases that had comorbid pathologies in the frontal cortex, such as beta-amyloid plaques, alpha-synuclein Lewy bodies or TDP43 inclusions. These are common pathologies observed in CTE cases [9, 14, 100] and may influence the pathogenesis of the disease. Our CTE group also contained a mixture of high- and low-severity cases and due to the availability of tissue, all the CTE cases were male. Lastly, to protect the anonymity of the subjects included in the study, we have not presented the cause of death information in the case demographics. However, as peripheral cancers and cardiovascular disease have been reported to affect glial reactivity and blood–brain barrier dysfunction, we have indicated cases where these conditions were noted as the cause of death. Overall, despite these limitations, our study provides new insights into the microenvironment of the CTE lesion and the potential role of perivascular glial reactivity in CTE pathogenesis. As global brain banking of CTE tissue continues to grow, we anticipate future studies will be able to address these limitations and elucidate the contribution of age, sex, and comorbid pathologies in the pathogenesis of the CTE p-tau lesions.

## Conclusion

In conclusion, we performed a comprehensive multiplex immunohistochemical analysis of the CTE pathognomonic lesion and identified glial reactivity as a prominent feature. This glial reactivity is largely characterised by astrocyte reactivity. We hypothesise that a population of L-ferritin+ NQO1+ astrocytes may arise as a compensatory response to mitigate iron-induced oxidative stress arising from a compromised blood–brain barrier. A priority for future studies will be to examine the integrity of the blood–brain barrier and investigate how neuroinflammation contributes to p-tau accumulation.

## Supplementary Information

Below is the link to the electronic supplementary material.

Supplementary file1 (DOCX 7334 KB)

Supplementary file2 (XLSX 17 KB)

## Data Availability

The data used for this study is available from the corresponding author upon reasonable request.

## Abbreviations

AD: Alzheimer’s disease
ALDH1L1: Aldehyde dehydrogenase 1 family member L1
AQP4: Aquaporin IV
ASBB: Australian Sports Brain Bank
CD68: Cluster of differentiation 68
CHI3L1: Chitinase-3 like-protein-1
CNPase: Cyclic nucleotide 3′-phosphodiesterase
CTE: Chronic traumatic encephalopathy
EDTA: Ethylenediaminetetraacetic acid
L-Ferritin/FTL: Ferritin light chain
GFAP: Glial fibrillary acidic protein
HLA-DR: Human leukocyte antigen DR
HuBB: Human Brain Bank
IBA1: Ionised calcium‐binding adaptor molecule 1
IHC: Immunohistochemistry
NFT: Neurofibrillary tangles
NINDS: National Institute of Neurological Disorders and Stroke
NQO1: NAD(P)H quinone dehydrogenase 1
PBS: Phosphate buffered saline
P2RY12: Purinergic receptor P2Y G protein-coupled 12
PMD: Postmortem delay
p-tau: Phosphorylated tau
TDP43: Transactive response DNA binding protein 43
UNITE: Understanding Neurological Injury and Traumatic Encephalopathy
