# Supplementary material for: Perivascular glial reactivity is a feature of phosphorylated tau lesions in chronic traumatic encephalopathy
Source: Acta Neuropathol. 2025 Feb 8;149(1):16. doi: 10.1007/s00401-025-02854-x (PMC11807024; doi:10.1007/s00401-025-02854-x)
Supplement: Supplementary file 1 — Supplementary file1 (DOCX 7334 KB) [file 401_2025_2854_MOESM1_ESM.docx]

Perivascular glial reactivity is a feature of phosphorylated tau lesions in Chronic Traumatic Encephalopathy

Chelsie Osterman^1^, Danica Hamlin^1^, Catherine M. Suter^2,3^, Andrew J. Affleck^2^, Brian S. Gloss^4^, Clinton P. Turner^1,5^, Richard L. M. Faull^1^, Thor Stein^,6,7,8,9^, Ann McKee^6,7,8,9^, Michael E. Buckland^2,3^, Maurice A. Curtis^1^, Helen C. Murray*^1^

**Affiliations**

^1^Department of Anatomy and Medical Imaging and Centre for Brain Research, Faculty of Medical and Health Science, University of Auckland, 85 Park Road, Grafton 1023, Auckland, New Zealand.

^2^Department of Neuropathology, Royal Prince Alfred Hospital, 94 Mallet St, Camperdown, NSW 2050, Australia.

^3^School of Medical Sciences, Faculty of Medicine and Health, University of Sydney, Camperdown, NSW 2006, Australia.

^4^Westmead Research Hub, Westmead Institute for Medical Research, Westmead, NSW, Australia

^5^Department of Anatomical Pathology, Pathology and Laboratory Medicine, Auckland City Hospital, 2 Park Road, Grafton 1023, Auckland, New Zealand.

^6^Department of Pathology and Laboratory Medicine, VA Boston Healthcare System, Boston, Massachusetts

^7^Department of Pathology, Boston University Chobanian & Avedisian School of Medicine, Boston, Massachusetts

^8^Boston University Alzheimer’s Disease and CTE Center, Boston University Chobanian & Avedisian School of Medicine, Boston, Massachusetts

^9^Bedford Veterans Affairs Medical Center, Bedford, Massachusetts

***Corresponding author:**

Helen Murray: [h.murray@auckland.ac.nz](mailto:h.murray@auckland.ac.nz)

**Supplementary Data**

Supplementary Figure 1: Overview of antibody panels and empirical validation of multiplex IHC labelling.

Supplementary Figure 2: Validation of multiplex labelling with single round labelling.

Supplementary Figure 3. Method for creating binary masks for measurement of percentage area of labelling.

Supplementary Figure 4: Overview of multiplex labelling illustrating axon and blood vessel markers in the frontal cortex of CTE cases.

Supplementary Figure 5: RNA and protein distribution for reactive gliosis markers in CTE lesion sulcus.

Supplementary Figure 6: Analysis of percentage marker area by CTE severity and age at death.


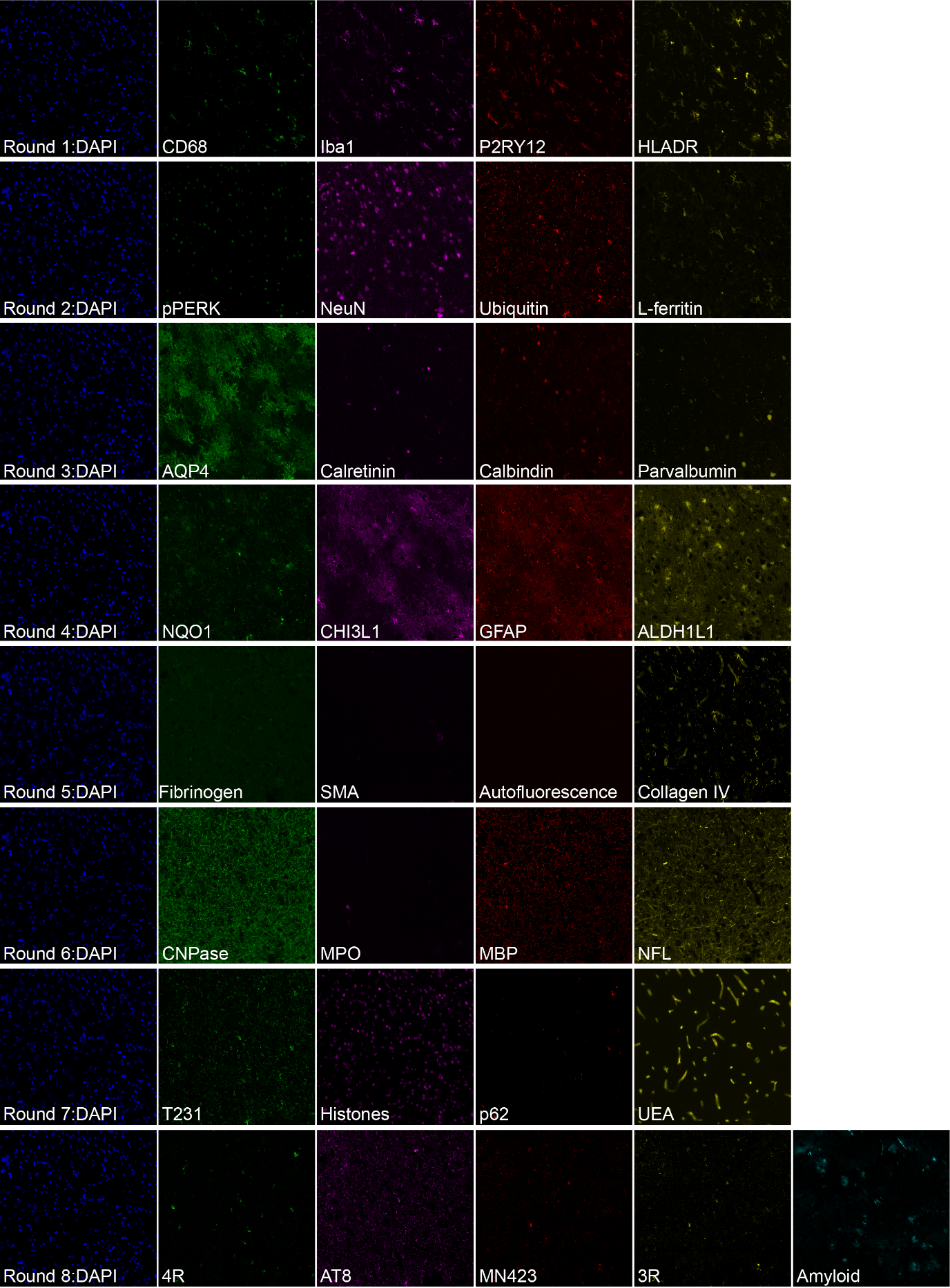


**Supplementary Figure 1. Overview of antibody panels and empirical validation of multiplex IHC labelling.** Example labelling of all antibodies included in this study on case AZ123. The labelling was empirically validated based on the unique design of the antibody panels, whereby, each primary antibody used in a round was selected from a different host species and/or immunoglobulin class/subclass and the antibodies selected for the subsequent round predominantly targeted a spatially distinct sub-cellular location or tissue structure. The primary antibodies were visualised using an appropriate secondary antibody conjugated to different spectrally non-overlapping fluorophores and the resulting fluorescence signal from each antibody was imaged in spectrally non-overlapping channels with minimal spectral crosstalk as previously reported (Maric et al., 2021). The results show no significant cross-reactivity between antibodies, nor spectral crosstalk between fluorescence signals in each imaging channel for each labelling round.


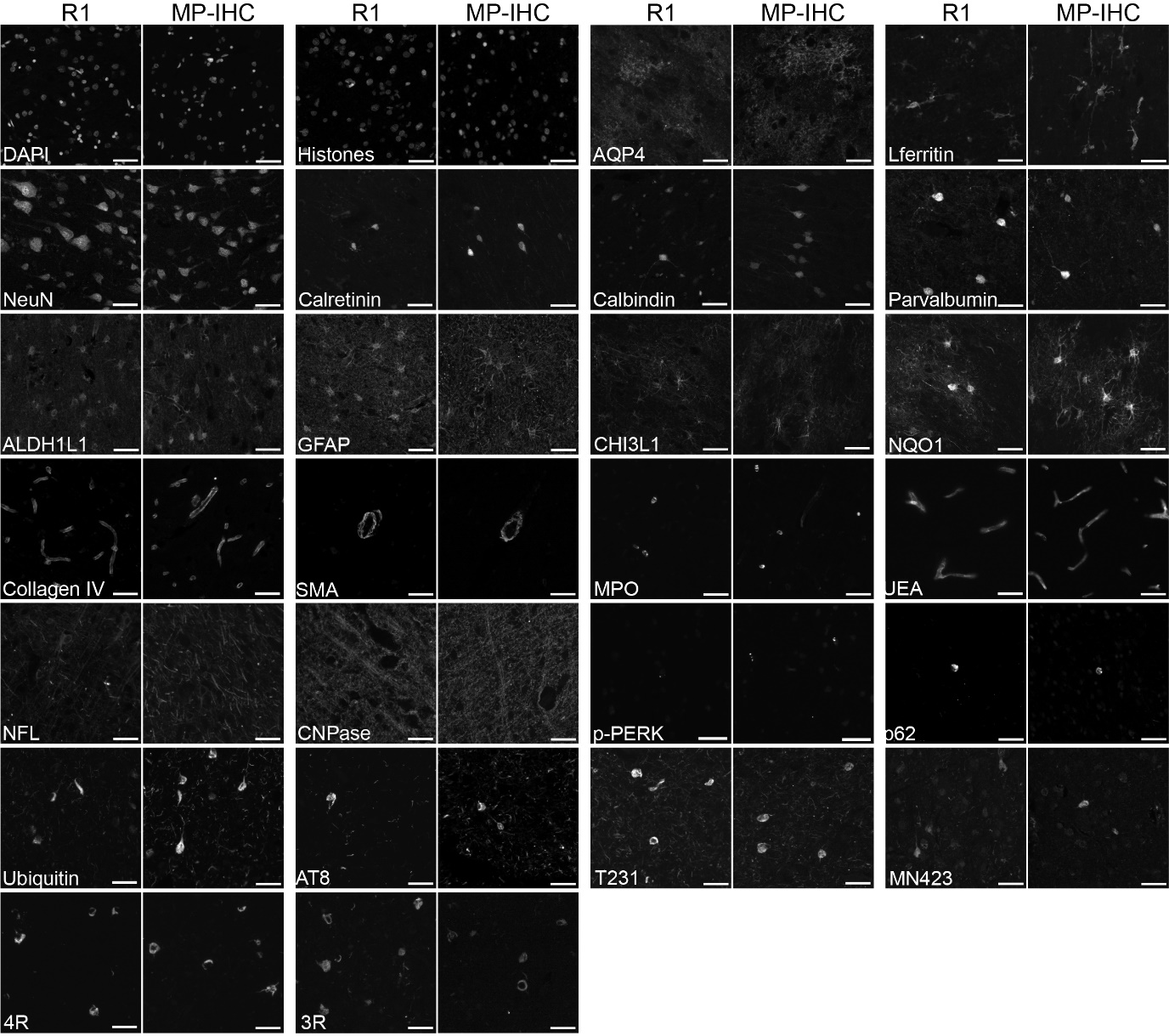


**Supplementary Figure 2. Validation of multiplex labelling with single round labelling.** Example images of each antibody used in this study as labelled using standard single-round fluorescent immunohistochemistry and in their designated panel for multiple-round multiplexed immunohistochemistry. Note that the first-round antibodies (Iba1, P2RY12, CD68, and HLADR) are not included here, as the multiplex labelling protocol for round 1 does not deviate from the standard single-round protocol. The results show no difference in the signal intensity or labelling distribution for any of the antibodies between standard or multiplexed labelling.


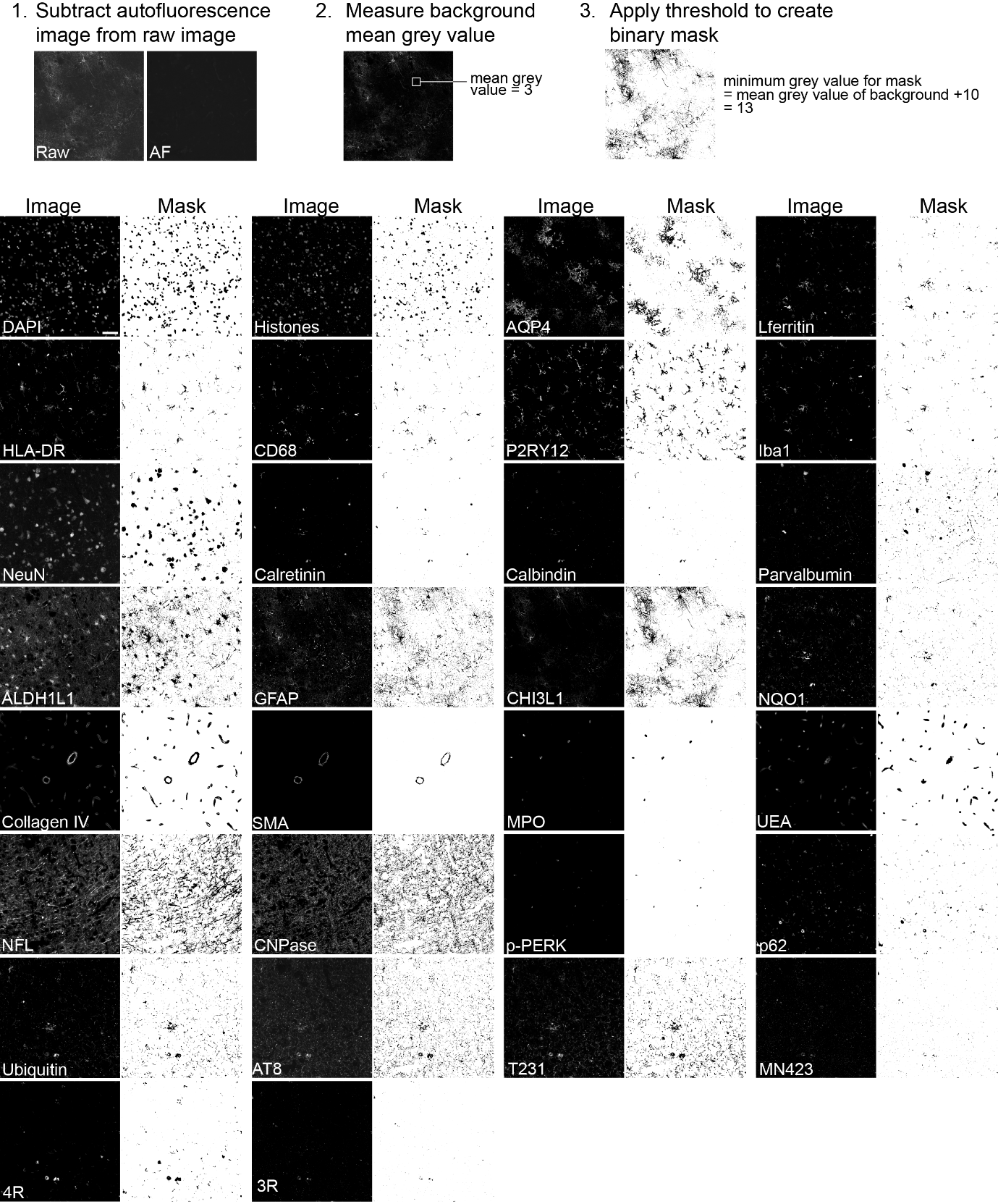


**Supplementary Figure 3. Method for creating binary masks for measurement of percentage area of labelling.** ImageJ was used for the binary mask steps **(a).** The autofluorescence channel image was first subtracted from each marker image in the stack. The mean grey value of the background staining was then measured in a 50 pixel^2^ area for each image. The threshold tool was then used to create binary masks of positive labelling for each marker. The lower threshold limit was set at 10 grey values above the measured background mean grey value, and the upper limit was set at 255, the maximum for 8-bit images. (b) Examples of the autofluorescence subtracted images, and the binary mask output are presented for each marker from one AD case, as all markers were expressed at reasonably high levels in AD cases.


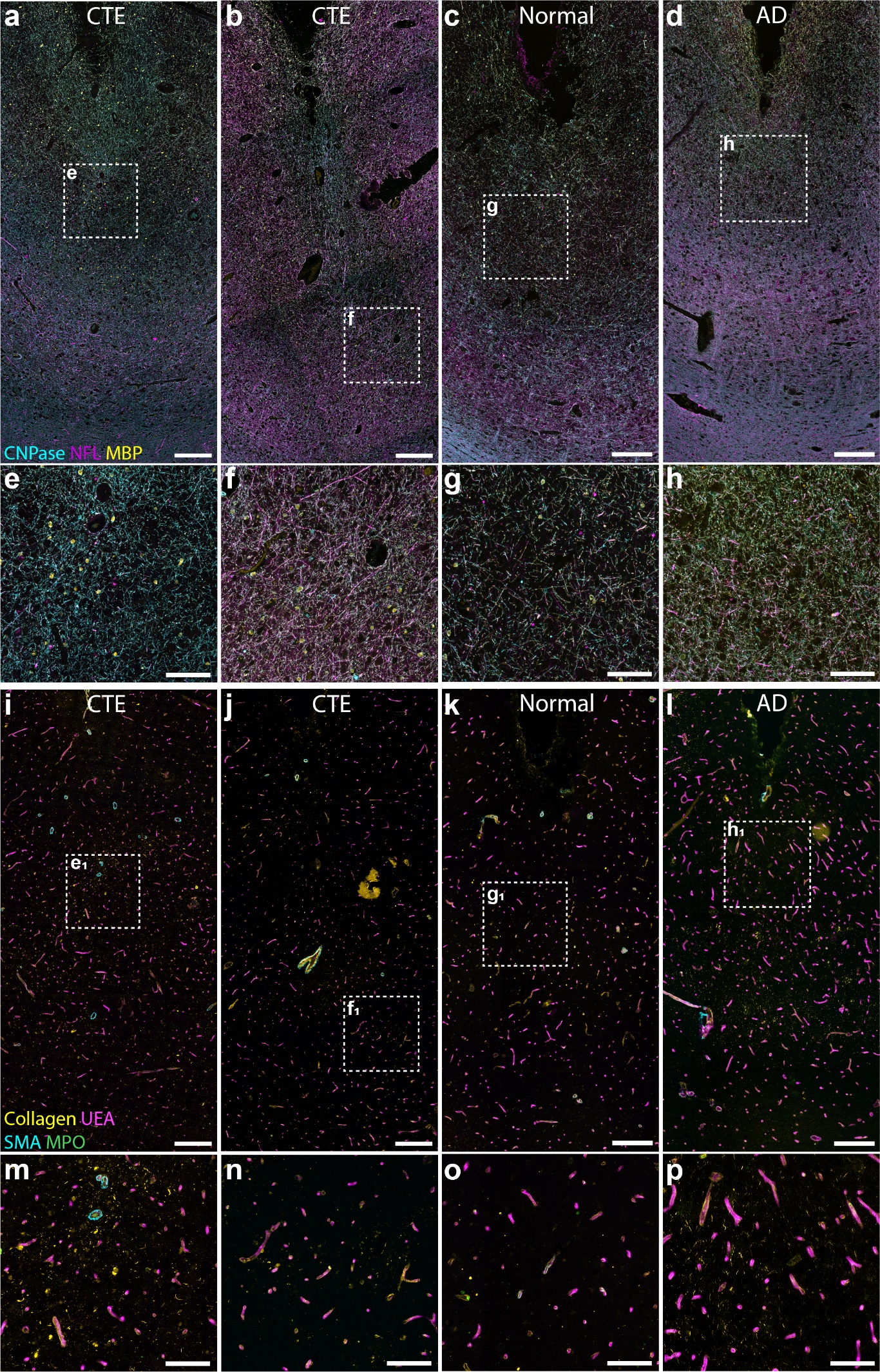


**Supplementary Figure 4: Overview of multiplex labelling illustrating axon and blood vessel markers in the frontal cortex of CTE cases.** Overview of the area at the depth of the cortical sulcus containing a tau lesion in two CTE cases from the Australia Sports Brain Bank **(a-b, i, j)**, and the corresponding area in a representative neurologically normal **(c, k)** and AD case **(d, l)**. Multiplex labelling for axon markers CNPase, neurofilament light (NFL), and myelin basic protein (MBP) are presented **(a-h)**, alongside a higher magnification of the lesion vessel and comparable regions in AD and normal cases indicated by the dotted box **(e-h)**. No overt differences in axon density are observed. Multiplex labelling for blood vessel markers collagen IV, UEA lection, smooth muscle actin (SMA) and myeloperoxidase (MPO) for neutrophils **(i-l)**, along with higher magnification of the regions indicated by the dotted box **(m-p)**. No difference in blood vessel density is qualitatively observed. Scale bars: 250 µm **(a-d, i-l)**; 100 µ, **(e-h, m-p)**.


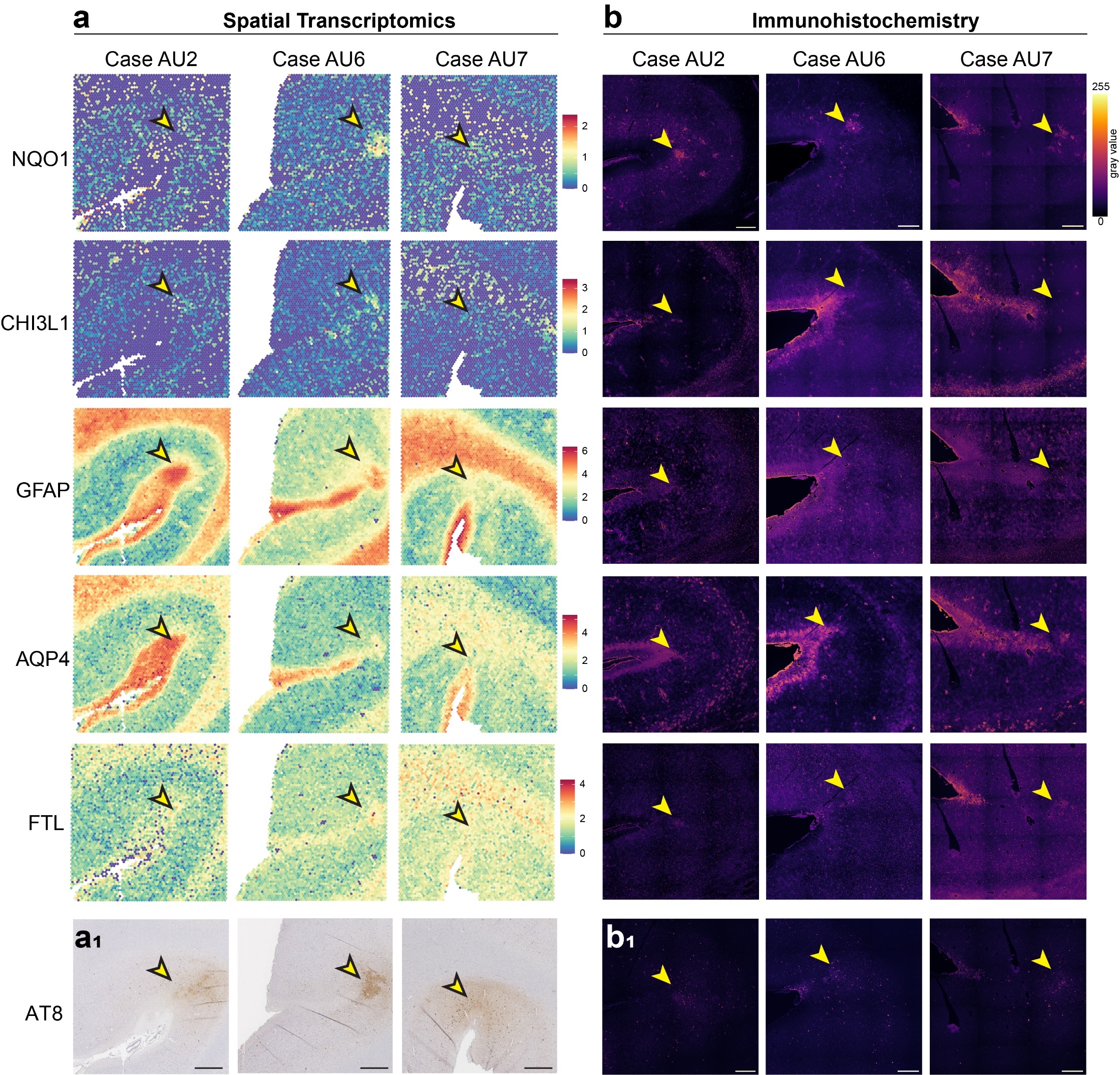


**Supplementary Figure 5: RNA and protein distribution for reactive gliosis markers in CTE lesion sulcus.** (a) Visium spatial transcriptomics illustrates the RNA distribution for NQO1, CHI3L1, GFAP, AQP4 and FTL (L-ferritin) within a lesion sulcus for three CTE cases from the Australia Sports Brain Bank. The phospho-tau (AT8) labelling for the same tissue sections is shown below each panel (a_1_). Scale bar: 200 µm. In all three cases, focal increases in NQO1, CHI3L1, GFAP, AQP4 and FTL (L-ferritin) RNA and protein expression are seen in cortical layer 1, the white matter and within the lesion area where AT8 tau (yellow arrow) is highly concentrated.


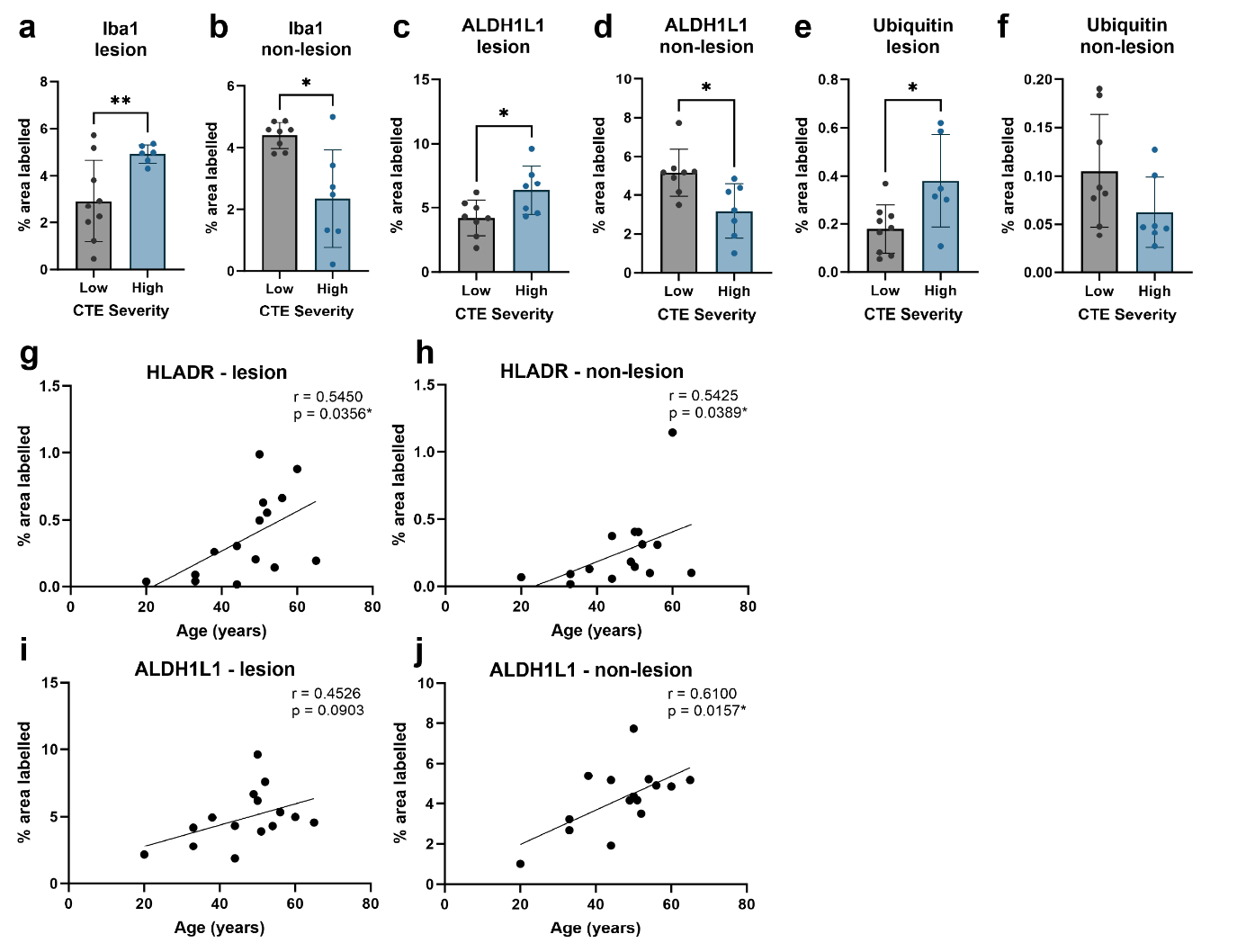


**Supplementary Figure 6: Analysis of percentage marker area by CTE severity and age at death.**  We investigated whether any of the markers we investigated differed between CTE cases classified as high and low severity. Interleaved scatter with bars plots shows the mean percentage area of labelling for each group ± standard deviation, with individual cases represented by each dot (**a-f**). There was a statistically significant increase in the percentage area labelled for Iba1 in the lesion area of high CTE cases compared to low cases (**a**, low CTE mean = 2.9 ± 1.7%, high CTE mean = 4.9 ± 0.4%; p = 0.0085, Unpaired t-test with Welch’s correction). Conversely, there was a statistically significant decrease in the percentage area labelled for Iba1 in the non-lesion area of high CTE cases compared to low CTE cases (**b**, low CTE mean = 4.4 ± 0.4%, high CTE mean = 2.4 ± 1.6%; p = 0.0135, Unpaired t-test with Welch’s correction). For ALDH1L1, the percentage area of labelling was significantly increased in the lesion area for high CTE cases (**c**, low CTE mean = 4.2 ± 1.4%, high CTE mean = 6.4 ± 1.9%; p = 0.0241, Unpaired t-test), however in non-lesion areas the percentage area was significantly decreased for high CTE cases (**d**, low CTE mean = 5.2 ± 1.2%, high CTE mean = 3.2 ± 1.4%; p = 0.0115, Unpaired t-test). For ubiquitin, the percentage area of labelling was significantly increased in the lesion area for high CTE cases (**e**, low CTE mean = 0.2 ± 0.1%, high CTE mean = 0.4 ± 0.2%; p = 0.0199, Unpaired t-test), but there was no significant difference between low and high CTE cases in the non-lesion areas (**f**). We also investigated whether the percentage area of labelling correlated with age at death for each of the markers we investigated. There was a significant positive correlation between age at death and the percentage area of labelling for HLADR in both lesion and non-lesion areas **(g,h**). There was also a significant positive correlation between age at death and the percentage area of labelling for ALDH1L1 in non-lesion areas (**j**), but not in lesion areas (**i**).
